# Soluble Toll-like receptor 2 is a biomarker for sepsis in critically ill patients with multi-organ failure within 12 h of ICU admission

**DOI:** 10.1186/s40635-016-0116-z

**Published:** 2017-01-13

**Authors:** Benjamin Holst, Tamas Szakmany, Anne-Catherine Raby, Vincent Hamlyn, Kimberley Durno, Judith E. Hall, Mario O. Labéta

**Affiliations:** 1 Department of Anaesthesia, Intensive Care and Pain Medicine, School of Medicine, Cardiff University, Heath Park, Cardiff, CF14 4XN UK; 2 ACT Directorate, Cwm Taf University Health Board, Llantrisant, CF72 8XR UK; 3 Anaesthetic Directorate, Aneurin Bevan University Health Board, Newport, NP20 5UB UK; 4 Division of Infection and Immunity, Cardiff University, School of Medicine, Heath Park, Cardiff, CF14 4XN UK

## Abstract

Soluble TLR2 levels are elevated in infective and inflammatory conditions, but its diagnostic value with sepsis-induced multi-organ failure has not been evaluated. 37 patients with a diagnosis of severe sepsis/septic shock (sepsis) and 27 patients with organ failure without infection (SIRS) were studied. Median (IQR) plasma sTLR2 levels were 2.7 ng/ml (1.4–6.1) in sepsis and 0.6 ng/ml (0.4–0.9) in SIRS p < 0.001. sTLR2 showed good diagnostic value for sepsis at cut-off of 1.0 ng/ml, AUC:0.959. We report the ability of sTLR2 levels to discriminate between sepsis and SIRS within 12 h of ICU admission in patients with multi-organ failure.

## Findings

The diagnosis of sepsis in ICU patients with organ failure is fraught with difficulty since both clinical signs and current diagnostic tests may be equivocal. Source control and timely antibiotic administration are crucial; thus, diagnostic tests that might enable early exclusion or positive identification of an infective etiology of critical illness are the focus of intensive research [1].

Toll-like receptor 2 (TLR2) is a member of a family of immune receptors which trigger pro-inflammatory responses following recognition of a range of microorganisms such as bacteria, viruses, and fungi. TLR2 is mainly expressed by myeloid cells and a soluble form (sTLR2), capable of down-modulating TLR-mediated pro-inflammatory responses, is released in response to microbial challenges [2]. Plasma levels of sTLR2 are elevated in a number of infective and inflammatory conditions [3] but its diagnostic value in ICU patients with sepsis-induced multi-organ failure has not been evaluated.

Following ethical approval by the South East Wales Research Ethics Committee (reference number 10WSE/421, June 2011) registration with the UK Clinical Research Network (UKCRN; cellular and biochemical investigations in sepsis, ID 11231) and written consent, 37 patients presenting to the Royal Glamorgan Hospital 10-bedded mixed medical/surgical ICU between January 2011 and March 2014 with a diagnosis of severe sepsis or septic shock according to the ESICM/SCCM consensus criteria [4] were enrolled within 24 h of the presumed onset of their illness, together with a cohort of 27 patients with organ failure due to dysregulated host response of non-infective origin (SIRS group). Patients in the SIRS group were enrolled when fulfilled two or more SIRS criteria, had documented organ dysfunction, but were not treated with antimicrobials for known or presumed infection. Blood was drawn within 12 h of ICU admission and plasma sTLR2 and C-reactive protein levels were determined by ELISA methods following the manufacturer’s instructions (R&D Systems, Minneapolis, MN, sTLR2 in normal serum: 0.575 ± 0.140 ng/ml; range 0.296–0.880).

For statistical analysis, chi-square test and Mann-Whitney *U* test—when the Kruskal-Wallis test rejected the null hypothesis of no differences between medians—was used as appropriate. We assessed correlations between parameters using the Spearman Rho’s correlation test. A result was considered to be significant at *p* < 0.05. Receiver operating characteristic (ROC) curves were plotted and the respective areas under the curve were calculated. Statistics were performed using the Statistical Program for Social Sciences (SPSS®) 20.0 software for Mac (Chicago, IL, USA).

**Fig. 1:**
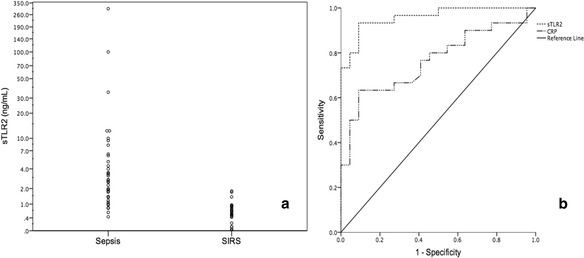
Individual sTLR2 levels in the sepsis and SIRS groups (**a**) and receiver operating characteristic curve (ROC) for the ability of sTLR2 and CRP levels to diagnose sepsis (**b**). Area under the curve (AUC) for sTLR2 0.959; 95% CI 0.912–1.000; AUC for CRP 0.764; 95% CI 0.635–0.894

Clinical characteristics of the patients in the sepsis and SIRS groups are presented in Table 1. Five patients in the sepsis group had culture-proven Gram-positive infection (four blood culture and one sputum), four had Gram-negative infection (all blood culture), and one had fungal infection (blood culture). Median (interquartile range) plasma sTLR2 levels were 2.7 ng/ml (1.4–6.1) in patients with severe sepsis and septic shock and 0.6 ng/ml (0.4–0.9) in patients with SIRS (*p* < 0.001 by Mann-Whitney *U* test, Fig. 1a). With a cut-off value of 1.0 ng/ml, sTLR2 showed good diagnostic value for sepsis, as it had a sensitivity of 90%, a specificity of 91%, and an AUC of 0.959 (Fig. 1b).

**Table 1:** Characteristics of patients in the sepsis and SIRS groups

	Sepsis *n* = 37	SIRS *n* = 27	*p* value
Age	63 (55–72)	63 (48–69)	0.298
Sex (M/F)	17/20	20/7	0.040
Admission SOFA	16 (14–19)	14 (13–17)	0.080
White cell count (10^9^/L)	15 (11–22)	13 (8–17)	0.169
Mean arterial pressure (mmHg)	78 (67–87)	79 (72–91)	0.445
PEEP (cm H_2_O)	8 (5–8)	7 (5–9)	0.892
SpO_2_ (%)	98 (95–100)	99 (98–100)	0.131
FiO_2_	0.5 (0.4–0.6)	0.4 (0.35–0.5)	0.326
Peak inspiratory pressure (cm H_2_O)	22 (19–25)	24 (16–29)	0.528
Creatinine (μmol/L)	158 (86–259)	88 (66–168)	0.010
Urea (mmol/L)	13.2 (8.6–18.3)	6.2 (4.2–10)	<0.001
Albumin (g/L)	20 (16–25)	31 (22–34)	<0.001
CRP (mg/L)	218 (129–340)	84 (40–168)	0.001
ICU stay (days)	10 (5–15)	4 (3–8)	0.016
Ventilated days	5 (2–9)	3 (1–4)	0.133
Shock days	3 (2–4)	0 (0–3)	0.002
Renal support days	0 (0–5)	0 (0–0)	0.011
In-hospital mortality	12 (32%)	11 (41%)	0.142

Data are presented as medians, with the interquartile ranges in *brackets*. For statistical analysis, Mann-Whitney *U* test and chi-square test were used to test differences between the sepsis and SIRS groups

In this feasibility pilot study, we report the ability of sTLR2 levels to discriminate between sepsis and SIRS within 12 h of ICU admission in a well-balanced group of patients with multi-organ failure. sTLR2 constitutes an important first-line negative regulatory mechanism to avoid harmful inflammatory responses [2]. The observed higher levels of plasma sTLR2 in sepsis (irrespective of the causative organism) than in SIRS patients suggest that this negative feedback mechanism is rapidly and preferentially activated upon infection. Notably, we found that sTLR2 has better specificity and sensitivity for sepsis than C-reactive protein, the most commonly used marker in the ICU (Fig. 1b, AUC - sTLR2 0.959; CRP - 0.764). It is encouraging that sTLR2 levels below 1 ng/mL had good ability to rule out sepsis whether it is secondary to bacterial or fungal infection within the first 12 h of ICU stay, when clinical parameters and traditional markers of infection are often equivocal. While sTLR2 levels on their own appear to be good markers of sepsis within the first 12 h of ICU admission, it is likely that they might perform even better as part of a biomarker panel [5]. Given the small sample size of our study, these promising findings warrant a larger confirmatory trial before sTLR2 levels can be introduced in the routine clinical practice.
